# Isolation, draft genome sequencing and identification of *Enterobacter roggenkampii* CCI9

**DOI:** 10.7150/jgen.46294

**Published:** 2021-01-01

**Authors:** Hironaga Akita, Yuya Itoiri, Noriyo Takeda, Zen-ichiro Kimura, Hiroyuki Inoue, Akinori Matsushika

**Affiliations:** 1 Research Institute for Sustainable Chemistry, National Institute of Advanced Industrial Science and Technology (AIST), 3-11-32 Kagamiyama, Higashi-Hiroshima, Hiroshima 739-0046, Japan.; 2 Department of Civil and Environmental Engineering, National Institute of Technology, Kure College, 2-2-11 Aga-minami, Kure, Hiroshima, 737-8506, Japan.; 3 Graduate School of Integrated Sciences for Life, Hiroshima University, 1-3-1 Kagamiyama, Higashi-Hiroshima, Hiroshima 739-8530, Japan.

**Keywords:** *Enterobacter*, Oligotroph, 16S rRNA, draft genome sequence, multilocus sequence analysis, average nucleotide identity

## Abstract

Strain CCI9, which was isolated from leaf soil collected in Japan, was capable of growth on poor-nutrient medium, at temperatures of 10°C to 45°C, at pHs of 4.5 to 10, and in the presence of 7.0% NaCl. We determined a draft genome sequence of strain CCI9, which consists of a total of 28 contigs containing 4,644,734 bp with a GC content of 56.1%. This assembly yielded 4,154 predicted coding sequences. Multilocus sequence analysis (MLSA) based on *atpD*, *gyrB*, *infB,* and *rpoB* gene sequences were performed to further identify strain CCI9. The MLSA revealed that strain CCI9 clustered tightly with *Enterobacter roggenkampii* EN-117^T^. Moreover, the average nucleotide identity value (98.6%) between genome sequences of strain CCI9 and *E. roggenkampii* EN-117^T^ exceeds the cutoff value for prokaryotic subspecies delineation. Therefore, strain CCI9 was identified as *E. roggenkampii* CCI9. To clarify differences between *E. roggenkampii* EN-117^T^ and CCI9, the coding proteins were compared against the eggNOG database.

## Introduction

Microbial bioproduction enables continuous production of desired substances from nutrients including sugars, nitrogens and minerals. In the present day, therefore, a variety of microorganisms are used industrially for production of enzymes 1, medicines 2, and organic acids 3. These microorganisms require high-nutrient media containing large amounts of sugar, nitrogen, phosphorus, mineral and other nutrients to optimize their productivities. On the other hand, the reduction of nutrient costs is important for achieving cost-effective production. Consequently, various attempts to reduce the production cost have been studied intensively in recent years. In general, the development of metabolically engineered strains with improved productivity of target substances is one of the most effective ways to reduce production costs 3. For example, an engineered *Mannheimia succiniciproducens* strain is shown to increase the productivity of succinic acid and to decrease the by-product formation 4. The utilization of agro-industrial wastes (such as sugarcane bagasse, potato peel waste and oil cake) as the nutrient sources also helps to reduce production costs 5.

Apart from those attempts, we expect that production costs could be reduced if an oligotrophic microorganism is used for industrial bioproduction, since oligotroph can grow under low-nutrient conditions. However, the doubling time of oligotroph is relatively long, and the growth of an obligate oligotroph is inhibited in the presence of high levels of nutrients 6. These limitations make oligotroph difficult to use for the industrial bioproduction. Thus, new oligotrophic strain with fast growth rates is needed. Here, we report the isolation of an oligotroph, strain CCI9, with a fast growth rate. To facilitate future in-depth genomic studies and industrial applications of this strain, strain CCI9 was subjected to genome sequencing.

## Materials and Methods

Soil samples were collected from Higashi-Hiroshima city in Hiroshima prefecture, Japan. A 1.5% agar plate (pH 7.2) without any additional carbon and nutrient sources was used for isolation of the objective oligotroph. Agar (Nacalai tesque, Kyoto, Japan) used in this study contains sulfate (<0.4%), calcium (<0.1%), iron, (<0.01%) and a few fatty acids and/or other minerals at concentrations less than 0.01%. The pH of agar plate was adjusted using NaOH. To isolate the oligotroph from the soil samples, soil samples were suspended at 10% (w/v) with sterilized water, and the suspension was then filtrated to remove the soil sample. Subsequently, the filtrate was inoculated onto the plate. After the plate was incubated overnight at 37°C, individual colony was successively re-streaked onto fresh 1.5% agar plates at least three times to obtain a pure colony.

After strain CCI9 was pre-grown overnight, the pre-culture was diluted to OD_600_ = 0.05 with fresh medium, the culture was incubated under several conditions. The OD_600_ was measured by monitoring the difference between cellular and cell-free turbidity values using an Eppendorf BioSpectrometer (Eppendorf, Hamburg, Germany). Growth of strain CCI9 was tested using Nutrient Broth (Kyokuto, Tokyo, Japan) at various temperatures (5-50°C) and pHs (4.0-11.0), and in the presence of various concentrations of NaCl (0-8.0%, w/v).

To prepare genomic DNA, strain CCI9 was cultured aerobically for 6 h at 37°C in Nutrient Broth (Kyokuto, Tokyo, Japan), and the cells were harvested by centrifugation. Extraction of genomic DNA from the cells was performed using an illustra^TM^ bacteria genomicPrep Mini Spin Kit (GE Healthcare, Chicago, IL, USA) according to the manufacturer's instructions. The concentration and purity of the resulting genomic DNA were measured using a QuantiFluor ONE dsDNA System (Promega, Madison, WI, USA) and NanoDrop ND-1000 spectrophotometer (Thermo Fisher Scientific, Waltham, MA, USA), respectively.

Genome sequencing was performed using a MiSeq (Illumina, San Diego, CA, USA) sequencer. Default parameters were used for all software unless otherwise specified. The *de novo* assembly of the raw data was carried out using A5-miseq ver.20160825 7. The tRNA and rRNA genes were detected using Prokka v.1.14.0 8. Genome annotation was performed using Prokka v.1.14.0 8 and the eggNOG-mapper website v.2.0.0 9. Average nucleotide identity (ANI) values were calculated by pairwise genome comparison of genome sequences of strain CCI9 and *E. roggenkampii* EN-117^T^ using the ANI algorithm 10 implemented within OrthoANIu tools 11. The Venn diagram based on the predicted coding sequences was constructed using OrthoVenn2 12. To clarify differences between *E. roggenkampii* EN-117^T^ and CCI9, the coding proteins were compared against the eggNOG database 9.

Multilocus sequence analysis (MLSA) was performed using the method of Brady et al. 13, 14. A phylogenetic tree of concatenated sequences (10,512 bp), including partial sequences of four housekeeping genes [*atpD* (encoding the β subunit of ATP synthase; 1383 bp),* gyrB* (encoding DNA gyrase; 2412 bp), *infB* (encoding translation initiation factor 2; 2688 bp), and *rpoB* (encoding the β subunit of RNA polymerase; 4029 bp)] from strain CCI9, was reconstructed using the maximum-likelihood method with the Tamura-Nei model 15. The housekeeping genes of the related type strains were obtained from the GenBank/DDBL/EMBL databases.

## Results and Discussion

**Figure 1:**
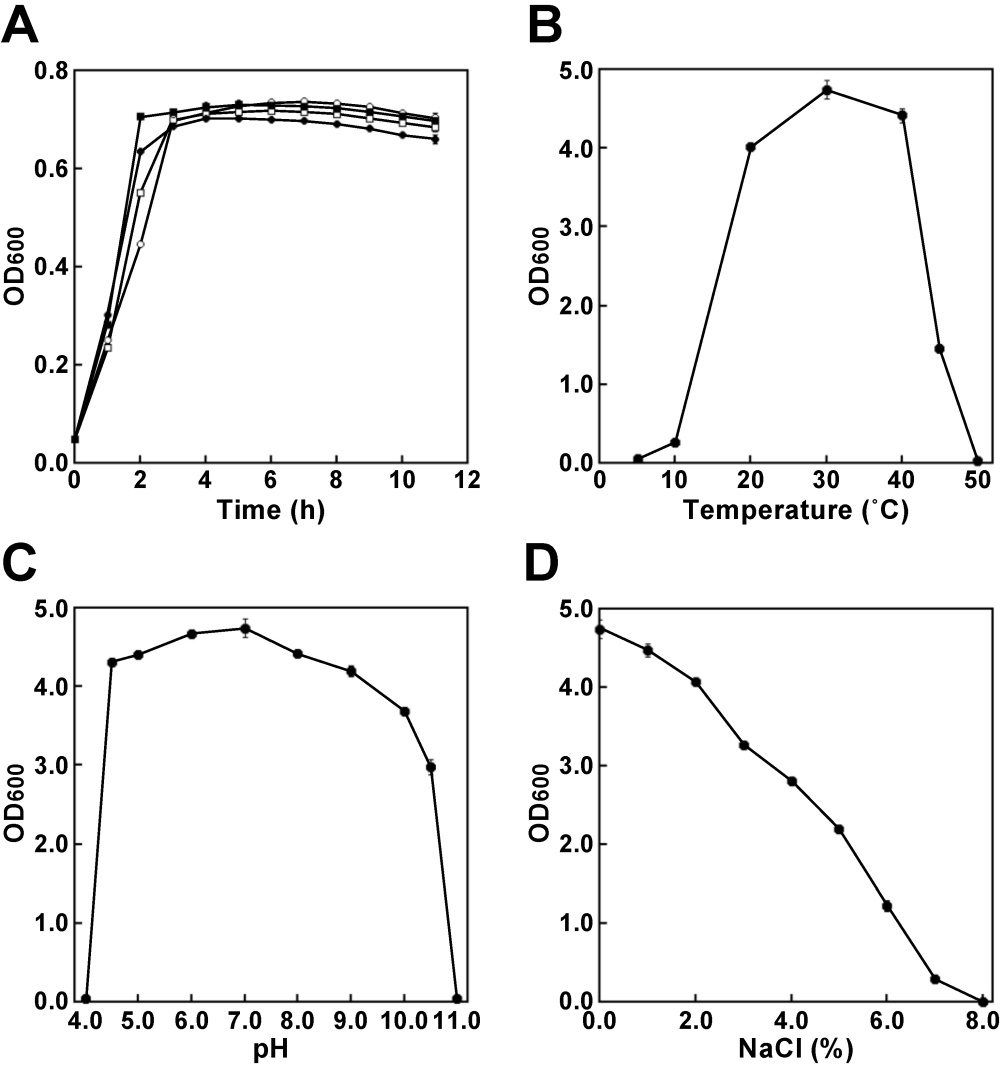
Phenotypic characterization of strain CCI9. **(A)** Growth ability at 37°C of strain CCI9 and* Escherichia coli* MG1655. The results for strain CCI9 and* E. coli* MG1655 are shown as filled and open symbols, respectively. The media are indicated as follows: circles, Nutrient Broth (pH 7.0) and squares, LB media (pH 7.0). The OD_600_ was measured using a Bio Microplate Reader HiTS (Scinics, Tokyo, Japan). **(B)** Effect of culture temperature on the growth ability of strain CCI9. Strain CCI9 was cultured at pH7.0.** (C)** Effect of culture pH on the growth ability of strain CCI9. Strain CCI9 was cultured at 30°C. **(D)** Effect of NaCl concentration on the growth ability of strain CCI9. Strain CCI9 was cultured at 30°C. Error bars indicate SE (*n* = 3).

To obtain oligotrophs, filtrates were prepared from several soil samples such as compost, leaf soil, mud and peat moss. Subsequently, the filtrates were plated onto 1.5% agar (pH 7.2) without an additive carbon source or other medium components. After incubation overnight at 37°C, a single colony was obtained from the filtrate of leaf soil, which is a soil composed mainly of decaying leaves. A purified colony was obtained through standard dilution plating on the same plates, and this isolate was designated strain CCI9 (strain number: HUT-8146). By contrast, *Escherichia coli* MG1655, which is used to create a bioproduction strain in many studies, did not grow on a 1.5% agar (pH 7.2). Although some oligotrophic bacteria are inhibited by high nutrient mixtures 16, 17, the growth of strain CCI9 was not inhibited in Nutrient Broth or LB medium. The growth rate of strain CCI9 was similar to that of *E. coli* MG1655, when cultured in Nutrient Broth or LB media (Fig. 1A). In Nutrient Broth, the strain CCI9 was capable of growing at temperatures of 10°C to 45°C (Fig. 1B) and at pHs of 4.5 to 10.5 (Fig. 1C), as well as showed the optimal growth at 30°C and pH 7.0. Moreover, this strain was tolerant to 7.0% (w/v) NaCl (Fig. 1D). These results suggested that we had successfully isolated the desired oligotroph.

To determine the taxonomy of strain CCI9, we determined a draft genome sequence of strain CCI9. The raw data from the MiSeq yielded 5,879,580 reads with 218-fold coverage. The assembled genome sequence of strain CCI9 consisted of 4,644,734 bp with a GC content of 56.1%. The 28 contigs in the assembly yielded the largest contig of 1,348,126 bp, and an N50 contig size of 283,167 bp. Within the genomic DNA of strain CCI9, 4,154 predicted coding sequences were identified, along with 83 tRNA genes and 11 rRNA genes.

**Figure 2:**
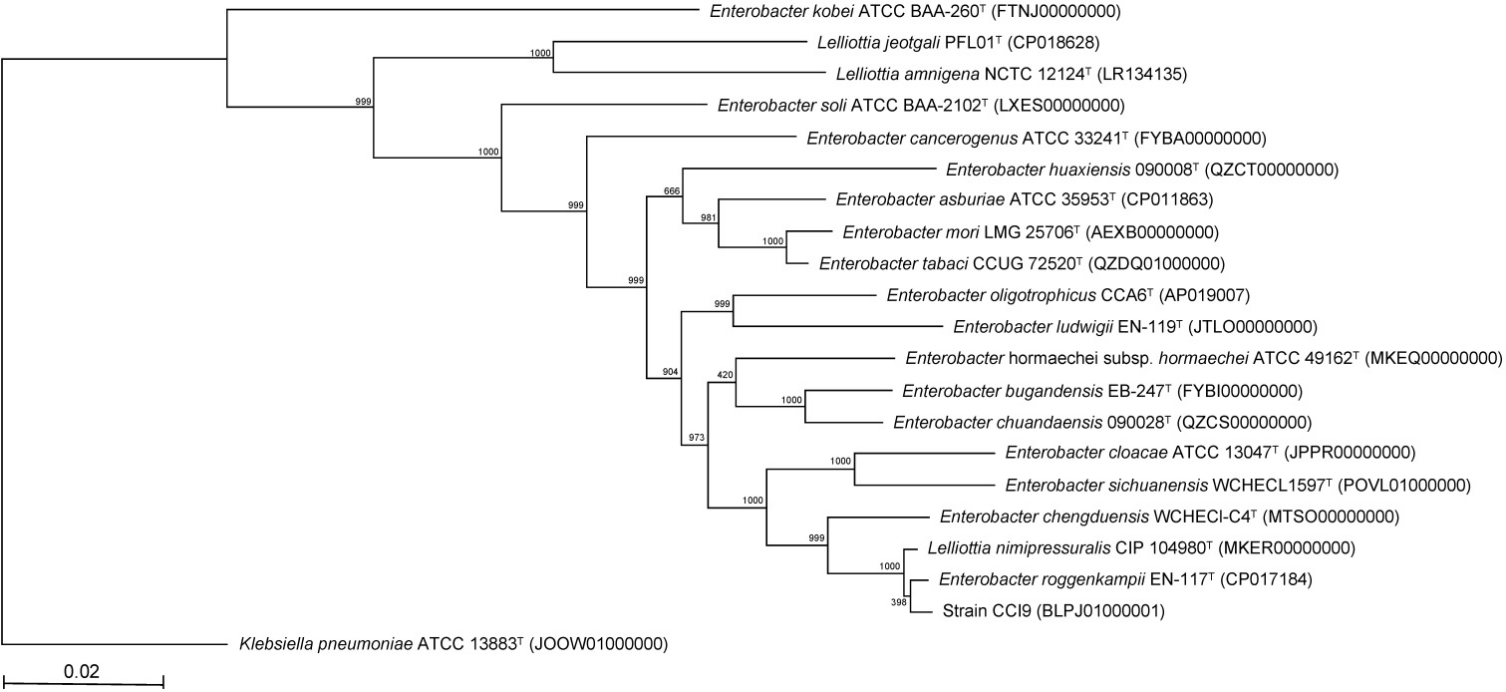
Phylogenetic tree constructed from analysis of the sequences of four housekeeping genes (*atpD*, *gyrB*, *infB*, and *rpoB*) and showing the relationships between strain CCI9 and the related *Enterobacter* type strains. The bar indicates a 0.02% nucleotide substitution rate. The tree was rooted using *Klebsiella pneumoniae aeruginosa* ATCC 13883^T^ as the outgroup.

As noted by Brady et al. 13, 14, MLSA is useful for determining the phylogeny of bacterial species. Therefore, we performed MLSA based on partial sequences of the *atpD*, *gyrB*, *infB*, and *rpoB* genes (Fig. 2). The resultant phylogenetic tree confirmed that strain CCI9 fell within a cluster comprising members of the genus *Enterobacter*. The most-closely related *Enterobacter* type strain was *E. roggenkampii* EN-117^T^ 18, with 99.5% sequence similarity. The genome completeness values of strain CCI9 and* E. roggenkampii* EN-117^T^ were 99.1%. The ANI value between strain CCI9 and* E. roggenkampii* EN-117^T^ was 98.6%, which exceeded the cutoff value of 98% for prokaryotic subspecies delineation 18. Thus, strain CCI9 was identified as *E. roggenkampii* CCI9.

**Figure 3:**
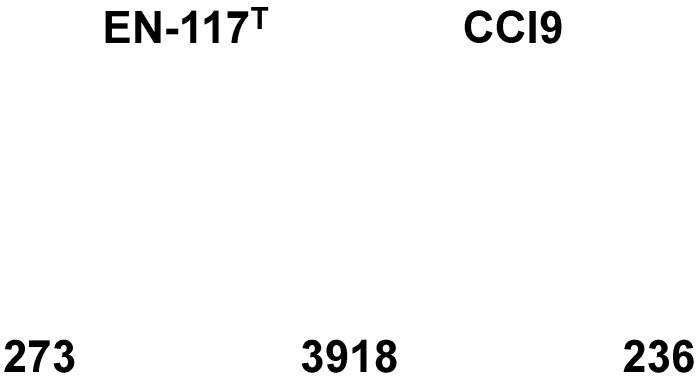
Venn diagram of the shared and unshared genes in *E. roggenkampii* EN-117^T^ and CCI9.

The Venn diagram shows 3918 common clusters of genes that show high similarity between the two *E. roggenkampii* strains (Fig. 3). On the other hand, 273 and 236 unique genes were present in the draft genome sequences of* E. roggenkampii* EN-117^T^ and CCI9, respectively. Among the eggNOG categories of both strains (Table 1), three kinds of genes involved in cell motility class (such as chemotaxis, fimbrial protein and outer membrane usher protein) were confirmed in the draft genome of *E. roggenkampii* CCI9. The motility properties of *E. roggenkampii* CCI9 may be different from that of *E. roggenkampii* EN-117^T^.

In this study, we described the isolation of strain CCI9, a bacterium capable of growth in nutrient-poor medium. After the draft genome sequence of strain CCI9 was determined, this isolate was identified as *E. roggenkampii* CCI9 based on MLSA and ANI value analysis. The draft genome sequence of* E. roggenkampii* CCI9 revealed their metabolic pathways, which is expected to use for the construction of engineered derivatives. Indeed, metabolically engineered *Enterobacter* strains for the production of acetoin have been constructed by inactivation of the by-product pathways 19, 20.

### Nucleotide Sequence Accession Number

The draft genome sequence of *E. roggenkampii* CCI9 has been deposited in the DDBJ/EMBL/GenBank databases under accession numbers BLPJ01000001 to BLPJ01000028. The raw sequence reads have been deposited in DDBJ under BioProject number PRJDB9407 and BioSample number SSUB014344.

## Figures and Tables

**Table 1:** eggNOG categories of coding proteins from unique genes in *E. roggenkampii* EN-117^T^ and CCI9

Class	Description	EN-117^T^		CCI9	
		Count	Proportion (%)	Count	Proportion (%)
	**Information storage and processing**				
J	Translation, ribosomal structure, and biogenesis	5	1.8	1	0.4
A	RNA processing and modification	0	0	0	0
K	Transcription	16	5.9	21	8.9
L	Replication, recombination, and repair	38	13.9	33	14.0
B	Chromatin structure and dynamics	0	0	0	0
	**Cellular processes and signaling**				
D	Cell cycle control, cell division, chromosome partitioning	0	0	0	0
Y	Nuclear structure	0	0	0	0
V	Defense mechanisms	6	2.2	8	3.4
T	Signal transduction mechanisms	1	0.4	6	2.5
M	Cell wall/membrane/envelope biogenesis	12	4.4	29	12.3
N	Cell motility	0	0	5	2.1
Z	Cytoskeleton	0	0	0	0
W	Extracellular structures	0	0	0	0
U	Intracellular trafficking, secretion, and vesicular transport	3	1.1	4	1.7
O	Posttranslational modification, protein turnover, chaperones	7	2.6	3	1.3
	**Metabolism**				
C	Energy production and conversion	7	2.6	5	2.1
G	Carbohydrate transport and metabolism	16	5.9	11	4.7
E	Amino acid transport and metabolism	7	2.6	6	2.5
F	Nucleotide transport and metabolism	2	0.7	1	0.4
H	Coenzyme transport and metabolism	2	0.7	2	0.8
I	Lipid transport and metabolism	2	0.7	2	0.8
P	Inorganic ion transport and metabolism	3	1.1	1	0.4
Q	Secondary metabolites biosynthesis, transport, and catabolism	1	0.4	3	1.3
	**Poorly characterized**				0
R	General function prediction only	0	0	0	0
S	Function unknown	145	53.1	95	40.3

## References

[1] Raveendran S, Parameswaran B, Ummalyma SB, Abraham A, Mathew AK, Madhavan A, Rebello S, Pandey A (2018). Applications of microbial enzymes in food industry. Food Technol Biotechnol.

[2] Jozala AF, Geraldes DC, Tundisi LL, Feitosa VA, Breyer CA, Cardoso SL, Mazzola PG, Oliveira-Nascimento L, Rangel-Yagui CO, Magalhães PO, Oliveira MA, Pessoa A Jr (2016). Biopharmaceuticals from microorganisms: from production to purification. Braz J Microbiol.

[3] Sauer M, Porro D, Mattanovich D, Branduardi P (2008). Microbial production of organic acids: expanding the markets. Trends Biotechnol.

[4] Lee SJ, Song H, Lee SY (2006). Genome-based metabolic engineering of *Mannheimia succiniciproducens* for succinic acid production. Appl Environ Microbiol.

[5] Sadh PK, Duhan S, Duhan JS (2018). Agro-industrial wastes and their utilization using solid state fermentation: a review. Bioresour Bioprocess.

[6] Ishida Y, Kadota H (1981). Growth patterns and substrate requirements of naturally occurring obligate oligotrophs. Microb Ecol.

[7] Tritt A, Eisen JA, Facciotti MT, Darling AE (2012). An integrated pipeline for *de novo* assembly of microbial genomes. PLoS ONE.

[8] Seemann T (2014). Prokka: rapid prokaryotic genome annotation. Bioinformatics.

[9] Huerta-Cepas J, Szklarczyk D, Heller D, Hernández-Plaza A, Forslund SK, Cook H, Mende DR, Letunic I, Rattei T, Jensen LJ, von Mering C, Bork P (2019). eggNOG 5.0: a hierarchical, functionally and phylogenetically annotated orthology resource based on 5090 organisms and 2502 viruses. Nucleic Acids Res.

[10] Goris J, Konstantinidis KT, Klappenbach JA, Coenye T, Vandamme P, Tiedje JM (2007). DNA-DNA hybridization values and their relationship to whole-genome sequence simi-larities. Int J Syst Evol Microbiol.

[11] Yoon SH, Ha SM, Lim J, Kwon S, Chun J (2017). A large-scale evaluation of algorithms to calculate average nucleotide identity. Antonie van Leeuwenhoek.

[12] Xu L, Dong Z, Fang L, Luo Y, Wei Z, Guo H, Zhang G, Gu YQ, Coleman-Derr D, Xia Q, Wang Y (2019). OrthoVenn2: a web server for whole-genome comparison and annotation of orthologous clusters across multiple species. Nucleic Acids Res.

[13] Brady C, Cleenwerck I, Venter S, Coutinho T, De Vos P (2013). Taxonomic evaluation of the genus *Enterobacter* based on multilocus sequence analysis (MLSA): proposal to reclassify *E. nimipressuralis* and *E. amnigenus* into *Lelliottia* gen. nov. as *Lelliottia nimipressuralis* comb. nov. and *Lelliottia amnigena* comb. nov, respectively, *E. gergoviae* and *E. pyrinus* into *Pluralibacter* gen. nov. as *Pluralibacter gergoviae* comb. nov. and *Pluralibacter pyrinus* comb. nov, respectively, *E. cowanii*, *E. radicincitans*, *E. oryzae* and *E. arachidis* into *Kosakonia* gen. nov. as *Kosakonia cowanii* comb. nov, *Kosakonia radicincitans* comb. nov, *Kosakonia oryzae* comb. nov. and *Kosakonia arachidis* comb. nov, respectively, and *E. turicensis*, *E. helveticus* and *E. pulveris* into *Cronobacter* as *Cronobacter zurichensis* nom. nov, *Cronobacter helveticus* comb. nov. and *Cronobacter pulveris* comb. nov, respectively, and emended description of the genera *Enterobacter* and *Cronobacter*. Syst Appl Microbiol.

[14] Brady C, Cleenwerck I, Venter S, Vancanneyt M, Swings J, Coutinho T (2008). Phylogeny and identification of *Pantoea* species associated with plants, humans and the natural environment based on multilocus sequence analysis (MLSA). Syst Appl Microbiol.

[15] Tamura K, Nei M (1993). Estimation of the number of nucleotide substitutions in the control region of mitochondrial DNA in humans and chimpanzees. Mol Biol Evol.

[16] Ohta H (2000). Growth characteristics of *Agromonas oligotrophica* on ferulic acid. Microbes Environ.

[17] Ohta H, Taniguchi S (1988). Growth characteristics of the soil oligotrophic bacterium *Agromonas oligotrophica* JCM 1494 on diluted nutrient broth. J Gen Appl Microbiol.

[18] Sutton GG, Brinkac LM, Clarke TH, Fouts DE (2018). *Enterobacter hormaechei* subsp. *hoffmannii* subsp. nov, *Enterobacter hormaechei* subsp. *xiangfangensis* comb. nov, *Enterobacter roggenkampii* sp. nov, and *Enterobacter muelleri* is a later heterotypic synonym of *Enterobacter asburiae* based on computational analysis of sequenced *Enterobacter* genomes. F1000Res.

[19] Jang JW, Jung HM, Im DK, Jung MY, Oh MK (2017). Pathway engineering of *Enterobacter aerogenes* to improve acetoin production by reducing by-products formation. Enzyme Microb Technol.

[20] Zhang L, Liu Q, Ge Y, Li L, Gao C, Xu P, Ma C (2016). Biotechnological production of acetoin, a bio-based platform chemical, from a lignocellulosic resource by metabolically engineered *Enterobacter cloacae*. Green Chem.

